# Intergenerational transmission of alcohol misuse: mediation and interaction by school performance in a Swedish birth cohort

**DOI:** 10.1136/jech-2019-213523

**Published:** 2020-06-10

**Authors:** Ylva B Almquist, Lauren Bishop, Nina-Katri Gustafsson, Lisa Berg

**Affiliations:** Department of Public Health Sciences, Stockholm University, Faculty of Social Sciences, Stockholm, Sweden

**Keywords:** Alcohol misuse, Intergenerational, School performance, Mediation, Interaction, Sweden

## Abstract

**Background:**

Children whose parents misuse alcohol have increased risks of own alcohol misuse in adulthood. Though most attain lower school marks, some still perform well in school, which could be an indicator of resilience with protective potential against negative health outcomes. Accordingly, the aim of this study was to examine the processes of mediation and interaction by school performance regarding the intergenerational transmission of alcohol misuse.

**Methods:**

Data were drawn from a prospective Swedish cohort study of children born in 1953 (n=14 608). Associations between parental alcohol misuse (ages 0–19) and participants’ own alcohol misuse in adulthood (ages 20–63) were examined by means of Cox regression analysis. Four-way decomposition was used to explore mediation and interaction by school performance in grade 6 (age 13), grade 9 (age 16) and grade 12 (age 19).

**Results:**

Mediation and/or interaction by school performance accounted for a substantial proportion of the association between parental alcohol misuse and own alcohol misuse in adulthood (58% for performance in grade 6, 27% for grade 9 and 30% for grade 12). Moreover, interaction effects appeared to be more important for the outcome than mediation.

**Conclusion:**

Above-average school performance among children whose parents misused alcohol seems to reflect processes of resilience with the potential to break the intergenerational transmission of alcohol misuse. Four-way decomposition offers a viable approach to disentangle processes of interaction from mediation, representing a promising avenue for future longitudinal research.

## INTRODUCTION

Substantial evidence shows that alcohol misuse is transmitted across generations.^1–^^3^ Common explanations for this transmission include genetic predispositions for alcoholism that are passed on from parents to children, environmental conditions such as prenatal exposure to alcohol and psychosocial difficulties arising from living with an alcoholic parent.^4 5^ Studies have also highlighted possible gene–environment interactions; for example, alcohol availability and witnessing episodes of drunkenness at home could trigger a genetic predisposition for alcoholism.^6–^^8^

With a strong focus on the family as the primary developmental context and the view of child agency as bounded in intergenerational relations, it might be easy to overlook the fact that a large part of children’s lives plays out in contexts outside the family, such as the school. Above-average school performance constitutes perhaps one of the most tangible protective resources derived from the school context. High school marks can be seen as a ﻿resource that enables children to reach higher levels of education, which makes them more competitive within the labour market, and further promotes healthy development across the life course.

Children with alcohol misusing parents tend to encounter problems in the school setting. Several studies have shown that children whose parents misuse alcohol are more likely to attain lower marks in school.^9 10^ Poor school performance, in turn, has been associated with an increased risk of alcohol misuse in adulthood.^11^ However, many children whose parents misuse alcohol still perform well in school. This might indicate that the child has developed agentic capacities, which allow them to further generate and capitalise on resources that promote health, even more so than peers who also perform well but have not experienced parental alcohol misuse. Such a situation—when a child ‘prevails’ over adversity—has in previous literature been assumed to reflect resilience.^12^

The role of school performance can be statistically explored using mediation and interaction, but traditional mediation methods are not well suited to disentangle the attributable proportionalities of each.^13^ Potential mediation effects (ie, having alcohol misusing parents increases the likelihood of attaining low school marks, which increases the risk for alcohol misuse in adulthood) and interaction effects (ie, above-average school performance may be even more protective against adult alcohol misuse for children whose parents misused alcohol) need to be assessed simultaneously to better interpret the total effect. This is possible with the newly developed four-way decomposition method.^14^

Based on a longitudinal investigation of a Swedish cohort born in 1953, the present study will first establish the overall association between parental alcohol misuse (age 0–19) and alcohol misuse in adulthood (ages 20–63). Then, we will decompose this association into mediation and interaction by school performance in grade 6 (age 13), grade 9 (age 16) and grade 12 (age 19). More specifically, we will use the four-way decomposition approach to answer the following questions:

- What is the risk of alcohol misuse in adulthood among individuals whose parents misused alcohol relative to individuals whose parents did not misuse alcohol (*total effect*)?
- What would be the risk of alcohol misuse in adulthood among individuals whose parents misused alcohol, relative to individuals whose parents did not misuse alcohol, if everyone had a high level of school performance (*neither mediation nor interaction*)?
- What is the combined risk of alcohol misuse in adulthood among individuals whose parents misused alcohol and who had a low level of school performance, if having alcohol misusing parents is not necessary for a low level of school performance (*interaction only*)?
- What is the combined risk of alcohol misuse in adulthood among individuals whose parents misused alcohol and who had a low level of school performance, if having alcohol misusing parents is necessary for a low level of school performance (*mediated interaction*)?
- What is the risk of alcohol misuse in adulthood among individuals who had a lower level of school performance, if having alcohol misusing parents is necessary for a low level of school performance (*mediation only*)?

## METHODS

### Data material

This study used the Stockholm Birth Cohort Multigenerational Study (SBC Multigen), a unique data material established in 2018/2019 through a probability matching of two anonymised, longitudinal data materials: The Stockholm Metropolitan Study (SMS) and RELINK53. The SMS is defined as all individuals born in 1953 who were living in the Stockholm metropolitan area in 1963 (n=15 117). Survey and register data were collected until 1986, after which the SMS was deidentified. RELINK53 is defined as everyone born in 1953 and residing in Sweden in 1960, 1965 and/or 1968, as well as their family linkages (parents, siblings, children, etc), comprising administrative register information between 1953 and 2018 for a total of 2 390 753 individuals. Using an algorithm based on variables identical to both data materials, 14 608 cohort members from the SMS were matched with RELINK53 and included in the SBC Multigen.^15^ The Stockholm Regional Ethical Review Board approved the creation of the SBC Multigen (no. 2017/34-31/5; 2017/684-32).

### Variables

Parental alcohol misuse information was drawn from local social registers (1953–1972, ages 0–19) based on records relating to paternal or maternal alcoholism (whether or not it resulted in institutional treatment or action by the temperance committee) and incidents of drunkenness (including misdemeanours and drunk driving). This measure has been used in several previous studies based on the same data material.^16 17^

Alcohol misuse in adulthood was operationalised as hospitalisations due to chronic and acute causes (100% attributable) of alcohol-related disease,^18^ indicated through records of in-patient care available through the National Patient Register (1973–2016, ages 20–63). The following ICD 10 codes were included: F10.0-F10.1 (alcohol abuse); F10.2 (alcohol dependence syndrome); F10.3-F10.9 (alcoholic psychosis); G31.2 (degeneration of nervous system due to alcohol); G62.1 (alcoholic polyneuropathy); G72.1 (alcoholic myopathy); I42.6 (alcoholic cardiomyopathy); K29.2 (alcoholic gastritis); K70.0-K.70.4, K70.9 (alcoholic liver disease); K86.0 (alcohol-induced chronic pancreatitis); X45, Y15, T51.0, T51.1, T51.9 (alcohol poisoning); X65 (suicide by and exposure to alcohol); and R78.0 (excessive blood level of alcohol). These codes were subsequently translated to the eighth and ninth revisions of the ICD.

Indicators of school performance were based on average school marks earned in grades 6 (upon completion of primary school, age 13/1966), 9 (upon completion of lower secondary school, age 16/1969) and 12 (upon completion of upper secondary school, age 19/1972). Information on school marks for grades 6 and 9 was collected from local registers kept by schools in the Stockholm region, whereas information concerning school marks in grade 12 was obtained from Statistics Sweden. During this period, marks in Sweden were given according to a 5-point (1–5) scale. The grading system was constructed to have a normal distribution at the national level, with a mean value of three and an SD of 1. In this study, the mean value was used as the cut-off to dichotomise the measures into ‘Above-average school marks’ and ‘At or below-average school marks’.

It is well established that men and individuals in lower socioeconomic positions have increased risks of alcohol misuse.^19–^^21^ Therefore, the following variables were included as possible confounders: gender, parental social class in 1953 (occupation of the head of the household), parental educational level in 1960 (number of adults in the household with a degree from upper secondary school) and parental income in 1964 (mean disposable income of the parents).

### Statistical analysis

Associations between parental alcohol misuse and alcohol misuse in adulthood—and mediation and interaction by school performance—were analysed using the *med4way* command in Stata. 15^22^ The *med4way* command requires fitting two regression models: Cox proportional hazards regression was specified to estimate the outcome (alcohol misuse in adulthood), which made it possible to take time to event into consideration. Subjects entered the study on 1 January 1973 and were censored on the date of the first hospitalisation due to alcohol misuse, on the date of death or at the end of follow-up (31 December 2016). A logistic model was specified for the mediator (school performance).

The *med4way* command reports on the overall association (‘Total effect’) as well as the four components into which it can be decomposed: controlled direct effect (‘Due to neither mediation nor interaction’), reference interaction (‘Due to interaction only’), mediated interaction (‘Due to mediated interaction’) and pure indirect effect (‘Due to mediation only’). Additional detail is provided in table 1. Moreover, we chose to include the full output in the regression tables, which includes the excess relative risks, effect risk ratios and overall attributable proportions. However, the discussion of the results does not focus on the excess relative risks.

**Table 1:** Definitions of the four-way decomposition as applied to the current study

Definition	Explanation	Application*
Total effect (*TE*)	*Total effect* Total effect of X (changing x* to x) on Y	What is the risk of alcohol misuse in adulthood among individuals whose parents misused alcohol relative to individuals whose parents did not misuse alcohol?
Controlled direct effect (*CDE*)	*Due to neither mediation nor interaction* Effect of X (changing x* to x) on Y, intervening to fix M to m	What would be the risk of alcohol misuse in adulthood among individuals whose parents misused alcohol, relative to individuals whose parents did not misuse alcohol, if everyone had a high level of school performance (ie, above-average school marks)?
Reference interaction (*INTref*)	*Due to interaction only* An additive interaction that operates only if M is present when X is x	What is the combined risk of alcohol misuse in adulthood among individuals whose parents misused alcohol and who had a low level of school performance (ie, at or below-average school marks), if having alcohol misusing parents is not necessary for a low level of school performance?
Mediated interaction (*INTmed*)	*Due to mediated interaction* An additive interaction that operates only if X (changing x* to x) has an effect on M	What is the combined risk of alcohol misuse in adulthood among individuals whose parents misused alcohol and who had a low level of school performance (ie, at or below average school marks), if having alcohol misusing parents is necessary for a low level of school performance?
Pure indirect effect (*PIE*)	*Due to mediation only* Effect of the mediator (changing m* to m) on Y when X is x, multiplied by the effect of X (changing x* to x) on M	What is the risk of alcohol misuse in adulthood among individuals who had a lower level of school performance, if having alcohol misusing parents is necessary for a low level of school performance (ie, at or below-average school marks)?

Y = Outcome (Alcohol misuse in adulthood).

X = Exposure (Parental alcohol misuse); x* = No; x = Yes.

M = Mediator (School performance); m* = At or below-average school marks; m = Above-average school marks.

*Here, we build on the definitions proposed by Bean *et al*.^23^

Two models were generated, of which the first (Model 1) was unadjusted and the second (Model 2) was adjusted for confounding by gender, parental social class, parental educational level and parental income.

Due to missing data, average school marks used in the decomposition by school performance were based on three samples: Sample A (grade 6, n=13 752), Sample B (grade 9, n=13 021) and Sample C (grade 12, n=6777). For Samples A and B, missing data were mainly due to cohort members moving and not having their school marks recorded in the local registers. For Sample C, missing data are almost exclusively due to cohort members not continuing to upper secondary school. Data were not missing for the other study variables.

Table 2 presents the descriptive statistics for the main study variables across the three samples.

**Table 2:** Descriptive statistics of the study variables

	Full sample n=14 608		Analytical sample A n=13 752		Analytical sample B n=13 021		Analytical sample C n=6777	
	n	%	n	%	n	%	n	%
**Parental alcohol misuse (ages 0–19)**								
No	13 703	94	12 906	94	12 299	94	6593	97
Yes	905	6	846	6	722	6	184	3
**Alcohol misuse in adulthood (ages 20–63)**								
No	13 858	95	13 056	95	12 426	95	6620	98
Yes	750	5	696	5	595	5	157	2
**School performance in grade 6 (age 13)**								
Above-average school marks			7407	54				
At or below-average school marks			6345	46				
**School performance in grade 9 (age 16)**								
Above-average school marks					6467	50		
At or below-average school marks					6554	50		
**School performance in grade 12 (age 19)**								
Above-average school marks							3352	49
At or below-average school marks							3425	51
**Gender (age 0)**								
Man	7447	51	6965	51	6506	50	3500	52
Woman	7161	49	6787	49	6515	50	3277	48
**Parental social class (age 0)**								
Working class/unclassified	7246	50	6898	50	6402	49	2350	35
Middle/upper middle class	7362	50	6854	50	6619	51	4427	65
**Parental educational level (age 7)**								
No adult in the household with a degree from upper secondary school/missing	10 847	74	10 256	75	9593	74	4089	60
At least one adult in the household with a degree from upper secondary school	3761	26	3496	25	3428	26	2688	40
**Parental income (age 11)**								
At or below average income/no registered income	8175	56	7666	56	7145	55	3029	45
Above-average income	6433	44	6086	44	5876	45	3748	55

## RESULTS

Table 3 presents the association between parental alcohol misuse and own alcohol misuse in adulthood, decomposed by school performance in grade 6. First, the unadjusted model (Model 1) shows that the risk of being hospitalised for alcohol misuse in adulthood is 2.76 times higher among individuals whose parents misused alcohol (‘Total effect relative risk ratio’, p=0.000). Moreover, 58% of the association (‘Overall proportion eliminated’) is due to mediation and/or interaction by school performance in grade 6. Interaction seems to be more important than mediation: 29% of the association is due to ‘interaction only’ versus 12% for ‘mediation only’. These figures increase to 46% and 28%, respectively, when also including ‘mediated interaction’ (denoted by ‘Overall proportion due to interaction’ and ‘Overall proportion due to mediation’). All estimates are statistically significant (p<0.05). In Model 2, the estimates are adjusted for gender, parental social class, parental educational level and parental income. The relative risk ratio for the association between parental alcohol misuse and own alcohol misuse in adulthood is reduced to 2.44 (p=0.000), whereas the figure for ‘Overall proportion eliminated’ drops to 45%. Interaction still seems to be more important than mediation—36% versus 23%—although the estimate for ‘Overall proportion due to interaction’ is no longer statistically significant (p=0.067).

**Table 3:** Parental alcohol misuse (ages 0–19) in relation to the risk of alcohol misuse in adulthood (ages 20–63). Results from Cox regression analysis with four-way decomposition by school performance in grade 6 (age 13). Based on Sample A (n=13 752)

	Alcohol misuse in adulthood (ages 20–63)					
	Model 1*			Model 2†		
	Estimate	P value	95% CI	Estimate	P value	95% CI
**Parental alcohol misuse (ages 0–19)**						
Total excess relative risk (*tereri*)	1.76	0.000	1.17, 2.34	1.44	0.000	0.84, 2.04
Four-way decomposition by school performance in grade 6 (age 13)						
Excess relative risk due to neither mediation nor interaction (*ereri_cde*)	0.76	0.032	0.17, 2.34	0.79	0.050	0.00, 1.58
Excess relative risk due to interaction only (*ereri_intref*)	0.74	0.025	0.07, 1.42	0.32	0.041	0.01, 0.62
Excess relative risk due to mediated interaction (*ereri_intmed*)	0.51	0.026	0.07, 0.96	0.19	0.046	0.04, 0.38
Excess relative risk due to mediation only (*ereri_pie*)	0.29	0.000	0.03, 0.54	0.14	0.000	0.09, 0.18
Total effect relative risk ratio (*tereria*)	2.76	0.000	2.17, 3.34	2.44	0.000	1.84, 3.04
Four-way decomposition by school performance in grade 6 (age 13)						
Proportion due to neither mediation nor interaction (*p_cde*)	0.42	0.019	0.07, 0.76	0.55	0.007	0.15, 0.95
Proportion due to interaction only (*p_intref*)	0.29	0.012	0.06, 0.52	0.22	0.067	−0.02, 0.46
Proportion due to mediated interaction (*p_intmed*)	0.16	0.013	0.03, 0.29	0.13	0.070	−0.01, 0.28
Proportion due to mediation only (*p_pie*)	0.12	0.000	0.07, 0.17	0.10	0.000	0.05, 0.14
Overall proportion due to mediation (*op_m*)	0.28	0.000	0.15, 0.42	0.23	0.007	0.06, 0.40
Overall proportion due to interaction (*op_ati*)	0.46	0.012	0.10, 0.81	0.36	0.067	−0.02, 0.73
Overall proportion eliminated (*op_e*)	0.58	0.001	0.22, 0.93	0.45	0.026	0.05, 0.85

*Unadjusted.

†Adjusted for gender, parental social class, parental educational level and parental income.

Component names, as specified in the *med4way* output, are presented in the table as italicised.

In table 4, the results for the four-way decomposition by school performance in grade 9 are presented. Model 1 shows that individuals whose parents misused alcohol are 2.54 times more likely to misuse alcohol in adulthood (‘Total effect relative risk ratio’, p=0.000). In total, 27% of the association is due to mediation and/or interaction (‘Overall proportion eliminated’). The proportion due to ‘interaction only’ is slightly higher than the proportion due to ‘mediation only’: 12% versus 10% (17% vs 15% when ‘mediated interaction’ is also included). However, while the estimate for ‘mediation only’ reaches a statistically significant level, the estimates for ‘interaction only’ and ‘mediated interaction’ do not. When adjusting for gender, parental social class, parental educational level and parental income in Model 2, the relative risk ratio for the association between parental alcohol misuse and own alcohol misuse in adulthood decreases to 2.49 (p=0.000). The ‘proportion eliminated’ by school performance in grade 9 is reduced to 17%.

**Table 4:** Parental alcohol misuse (ages 0–19) in relation to the risk of alcohol misuse in adulthood (ages 20–63). Results from Cox regression analysis with four-way decomposition by school performance in grade 9 (age 16). Based on Sample B (n=13 021)

	Alcohol misuse in adulthood (ages 20–63)					
	Model 1*			Model 2†		
	Estimate	P value	95% CI	Estimate	P value	95% CI
**Parental alcohol misuse (ages 0–19)**						
Total excess relative risk (*tereri*)	1.54	0.000	0.92, 2.17	1.49	0.000	0.77, 2.20
Four-way decomposition by school performance in grade 9 (age 16)						
Excess relative risk due to neither mediation nor interaction (*ereri_cde*)	0.12	0.011	0.25, 1.99	1.23	0.016	0.23, 2.23
Excess relative risk due to interaction only (*ereri_intref*)	0.19	0.511	−0.38, 0.76	0.11	0.577	−0.28, 0.49
Excess relative risk due to mediated interaction (*ereri_intmed*)	0.08	0.511	−0.15, 0.31	0.05	0.579	−0.11, 0.20
Excess relative risk due to mediation only (*ereri_pie*)	0.16	0.000	0.11, 0.20	0.10	0.000	0.06, 0.15
Total effect relative risk ratio (*tereria*)	2.54	0.000	1.92, 3.17	2.49	0.000	1.77, 3.20
Four-way decomposition by school performance in grade 9 (age 16)						
Proportion due to neither mediation nor interaction (*p_cde*)	0.73	0.004	0.23, 1.22	0.83	0.000	0.43, 1.22
Proportion due to interaction only (*p_intref*)	0.12	0.499	−0.23, 0.48	0.07	0.594	−0.20, 0.34
Proportion due to mediated interaction (*p_intmed*)	0.05	0.500	−0.09, 0.19	0.03	0.595	−0.08, 0.14
Proportion due to mediation only (*p_pie*)	0.10	0.000	0.05, 0.15	0.07	0.001	0.03, 0.11
Overall proportion due to mediation (*op_m*)	0.15	0.042	0.01, 0.30	0.10	0.133	−0.03, 0.23
Overall proportion due to interaction (*op_ati*)	0.17	0.499	−0.33, 0.67	0.10	0.594	−0.28, 0.48
Overall proportion eliminated (*op_e*)	0.27	0.279	−0.22, 0.77	0.17	0.390	−0.22, 0.57

*Unadjusted.

†Adjusted for gender, parental social class, parental educational level and parental income.

Component names, as specified in the *med4way* output, are presented in the table as italicised.

Table 5 shows the results for the decomposition by school performance in grade 12. In this positively selected sample of individuals who continued to upper secondary school, those whose parents misused alcohol have a 2.80 times higher risk of own alcohol misuse in adulthood (‘Total effect relative risk ratio’, p=0.001). Moreover, 70% of the association between parental alcohol misuse and alcohol misuse in adulthood remains after adjusting for school performance, whereas 30% is due to mediation and/or interaction. While interaction seems to be much more important than mediation (21% for ‘interaction only’ vs 3% for ‘mediation only’ and 27% vs 9% when ‘mediated interaction’ is also included), none of the estimates reaches statistical significance. Regarding the estimates in Model 2, the association between parental alcohol misuse and own alcohol misuse in adulthood is reduced to a relative risk ratio of 2.45 (p=0.002). While interaction still seems to be more important than mediation, none of the estimates are statistically significant.

**Table 5:** Parental alcohol misuse (ages 0–19) in relation to the risk of alcohol misuse in adulthood (ages 20–63). Results from Cox regression analysis with four-way decomposition by school performance in grade 12 (age 19). Based on Sample C (n=6777)

	Alcohol misuse in adulthood (ages 20–63)					
	Model 1*			Model 2†		
	Estimate	P value	95% CI	Estimate	P value	95% CI
**Parental alcohol misuse (ages 0–19)**						
Total excess relative risk (*tereri*)	1.80	0.040	0.08, 3.53	1.45	0.065	−0.09, 3.00
Four-way decomposition by school performance in grade 12 (age 19)						
Excess relative risk due to neither mediation nor interaction (*ereri_cde*)	1.26	0.290	−1.07, 3.61	1.20	0.313	−1.13, 3.53
Excess relative risk due to interaction only (*ereri_intref*)	0.38	0.648	−1.24, 2.01	0.19	0.806	−1.32, 1.69
Excess relative risk due to mediated interaction (*ereri_intmed*)	0.10	0.650	−0.34, 0.54	0.03	0.807	−0.23, 0.29
Excess relative risk due to mediation only (*ereri_pie*)	0.06	0.028	0.01, 0.11	0.03	0.123	−0.01, 0.07
Total effect relative risk ratio (*tereria*)	2.80	0.001	1.08, 4.53	2.45	0.002	0.91, 4.00
Four-way decomposition by school performance in grade 12 (age 19)						
Proportion due to neither mediation nor interaction (*p_cde*)	0.70	0.215	−0.41, 1.81	0.83	0.186	−0.40, 2.05
Proportion due to interaction only (*p_intref*)	0.21	0.638	−0.67, 1.09	0.13	0.807	−0.91, 1.17
Proportion due to mediated interaction (*p_intmed*)	0.06	0.640	−0.18, 0.29	0.02	0.808	−0.16, 0.20
Proportion due to mediation only (*p_pie*)	0.03	0.128	−0.01, 0.07	0.02	0.225	−0.01, 0.06
Overall proportion due to mediation (*op_m*)	0.09	0.465	−0.15, 0.33	0.04	0.648	−0.14, 0.23
Overall proportion due to interaction (*op_ati*)	0.27	0.638	−0.84, 1.38	0.15	0.807	−1.07, 1.37
Overall proportion eliminated (*op_e*)	0.30	0.597	−0.81, 1.41	0.17	0.781	−1.05, 1.40

*Unadjusted.

†Adjusted for gender, parental social class, parental educational level and parental income.

Component names, as specified in the *med4way* output, are presented in the table as italicised.

## DISCUSSION

This study demonstrated that a substantial proportion of the association between parental alcohol misuse and own alcohol misuse in adulthood was explained by mediation and/or interaction by school performance. Overall, interaction appeared to be more important than mediation, suggesting that while children living with alcohol misusing parents are more likely to perform worse in school and have subsequently higher risks of misusing alcohol in adulthood (ie, mediation), above-average performance among this group of children seems to mitigate the intergenerational transmission of alcohol misuse (ie, interaction). This might reflect resilience processes expanding from the school context. The significance of school performance was particularly evident in grade 6, and much weaker in grades 9 and 12. Although it could be interpreted that school performance at earlier ages is relatively more important compared with performance at later ages, it may also be an artefact created by the increasing positive selection across samples.

While this study is, to the best of our knowledge, the first to disentangle the role of school performance for the intergenerational transmission of alcohol misuse, the results are largely in line with previous research examining related questions. For example, an earlier study based on the SBC Multigen found that above-average school performance counteracted the increased risks for premature mortality among individuals with childhood experiences of out-of-home care.^24^ Another study, drawing on information from the Northern Swedish cohort, found that adversity during adolescence was less strongly associated with having poorer self-rated health in midlife among individuals who had an advantaged situation with regard to school, peers or spare time.^25^

The four-way decomposition analysis builds on a counterfactual or potential outcomes framework and requires that all confounders are controlled for.^22^ This may present some difficulties in the current study, especially since the measurements extend over long time periods; for example, factors that could confound the mediator–outcome association could very well be mediators for the exposure-outcome association. To reduce model complexity and avoid overadjustment, gender, parental social class, parental educational level and parental income were the only confounders included. While these adjustments did not significantly affect the association between parental alcohol misuse and own alcohol misuse in adulthood, they explained a rather substantial part of the mediation and interaction by school performance. Additional analyses (results not presented) showed that it was primarily the inclusion of parental educational level that led to the reduction of the estimates; this is not surprising given the strong intergenerational transmission of educational outcomes.^26 27^

### Strengths and limitations

Major strengths of this study included the prospective design, use of register data, long-term follow-up of alcohol misuse among parents and their adult children and multiple measurements of school performance. The large sample size rendered it possible to decompose the association between parental alcohol misuse and alcohol misuse in adulthood into mediation and interaction by school performance. Some limitations should nevertheless be addressed. One issue relates to the measurement period for parental alcohol misuse. In its original form, the information available in the social registers was divided into three periods. We combined these periods for the purposes of the current study, although this means that in some cases, the measure of school performance precedes the records of parental alcohol misuse. Nonetheless, since our indicator of parental alcohol misuse reflects a high degree of severity, it is unlikely there was no problematic use of alcohol before any documented consequences in the registers. Another limitation concerns the use of inpatient care data to indicate the cohort members’ own alcohol misuse. These data only capture the most severe cases, so a proportion of individuals are likely misclassified regarding the outcome. Finally, the measures of school performance were dichotomised using the mean value as the cut-off. Though it was possible to operationalise school marks as continuous factors or to dichotomise them differently, the mean value constitutes a conceptually clear distinction between low and high performance, which should be more relevant for policy.

The current study was based on a Swedish cohort who were born in the 1950s and attended school in the 1960s, which affects the generalisability of the findings. Nevertheless, there is nothing that suggests a weakening of the intergenerational transmission of alcohol misuse^28^ or the importance of school performance over time. The fact that we found these results for Sweden, which is generally considered to be a generous welfare state with well-developed social policies and free education,^29^ could indicate that the patterns might be even stronger in other countries.

## CONCLUSIONS

Above-average school performance, as a resource derived from the school setting, could potentially break (part of) the intergenerational transmission of alcohol misuse. Promoting academic achievement among children who have experienced adversities such as parental alcohol misuse should, therefore, be prioritised in relation to preventive efforts. Here, it is also necessary to consider how the socioeconomic living conditions of the children—particularly as reflected in the parents’ educational level—might influence the possibility to do so.

The findings from the current study highlight the need for further inquiry into the details of how resilience processes operate to counteract negative life trajectories. In this context, four-way decomposition seems to offer a viable approach to simultaneously address interaction and mediation, representing a promising avenue for future longitudinal research.

**What is already known on this subject**

- Children whose parents misuse alcohol have increased risks of alcohol misuse in adulthood. They generally also achieve lower school marks, which in turn is linked to increased risks of subsequent alcohol misuse. However, it is also clear from previous research that many children whose parents misuse alcohol still perform well in school, which could potentially counteract any negative consequences.

**What this study adds**

- While children whose parents misused alcohol are more likely to perform at or below average in school and have subsequently higher risks of misusing alcohol in adulthood, above-average performance among these children seems to reflect processes of resilience with the potential of breaking this association. Four-way decomposition offers a viable approach to disentangle processes of interaction from mediation, representing a promising avenue for future longitudinal research.
